# Potential of Activin B as a Clinical Biomarker in Myalgic Encephalomyelitis/Chronic Fatigue Syndrome (ME/CFS)

**DOI:** 10.3390/biom11081189

**Published:** 2021-08-11

**Authors:** Sabine Gravelsina, Zaiga Nora-Krukle, Anda Vilmane, Simons Svirskis, Katrine Vecvagare, Angelika Krumina, Modra Murovska

**Affiliations:** 1 Institute of Microbiology and Virology, Rīga Stradiņš University, 5 Ratsupites Str., LV-1067 Riga, Latvia; zaiga.nora@rsu.lv (Z.N.-K.); anda.vilmane@rsu.lv (A.V.); ssvirskis@latnet.lv (S.S.); katrine.vecvagare@rsu.lv (K.V.); modra.murovska@rsu.lv (M.M.); 2 Department of Infectology, Riga Stradins University, LV-1007 Riga, Latvia; angelika.krumina@rsu.lv

**Keywords:** ME/CFS, human activin B, visual analogue scale

## Abstract

Reliable serum biomarkers are of immense need for diagnostic purposes of myalgic encephalomyelitis/chronic fatigue syndrome (ME/CFS)—a disabling and complex disease for which diagnosis is mainly based on clinical symptoms. The aim of this study was to evaluate a possible diagnostic potential of activin B by directly comparing 134 cases of ME/CFS with 54 healthy controls. Analyses of human activin B level in plasma samples were performed using a validated human activin B ELISA assay. The results of the study show that activin B levels did not differ statistically significantly between ME/CFS patients and healthy controls (*p* = 0.6511). No gender or age-related differences in activin B levels were observed in the ME/CFS group and healthy controls. The level of activin B tended to decrease with increasing visual analogue scale score (*r* = −0.2004; *p* = 0.5085) nevertheless the results obtained so far does not support the clinical utility of activin B as a biomarker for ME/CFS.

## 1. Introduction

Myalgic encephalomyelitis/chronic fatigue syndrome (ME/CFS) is a complicated, chronic disease mainly characterized by severe fatigue with many clinical symptoms related to autonomic nervous system imbalance, cognitive impairment, immune and endocrine dysfunction [1]. The prevalence of ME/CFS worldwide varies from 0.1% up to 2.2% depending on the applied diagnostic criteria [2,3,4]. However, there are no European-wide estimates of disease burden [5].

The disease affects all ages, races and socioeconomic groups and some studies show that approximately three to four times as many women as men present with symptoms [6]. Despite many years of molecular and clinical research worldwide, there is still no unified definition for this heterogeneous disease. Another aspect of the complexity of ME/CFS is that no objective parameters or diagnostic markers exist to ensure an exact clinical assessment of the patient. However, the most widely used clinical definitions in clinical research are the Fukuda criteria and the International Consensus criteria, both of which demonstrate an inability to separate ME from CFS [7,8]. Genetic predisposition, stress, trauma, exposure to toxins, physical activity and rest ratio, as well as viral infections have been considered as potential etiological factors for ME/CFS [9,10]. The disease is mainly characterized by severe fatigue, post-exertional malaise, un-refreshing sleep, memory loss, difficulty concentrating, sore throat, lymphadenopathy, muscle pain and headaches. The pathomechanisms of ME/CFS are still under investigation, and there are no standardized biological markers or tests for diagnostics; therefore, even the existence of this medical diagnosis has been questioned for a long time [10,11,12]. The pathogenesis of ME/CFS is likely multi-factorial and various microbial and viral infections are possible trigger factors of ME/CFS. Still, a single infectious representative has not yet been confirmed and the role of viral infections in ME/CFS remains obscure as there is no proven correlation between ME/CFS severity and the stage of infectious process yet.

It is extremely important to develop simpler diagnostic tools from routine data to assist health professionals to diagnose ME/CFS and to monitor therapeutic approaches. Examination of the diagnostic potential of serum biomarkers would allow for the stratification of ME/CFS patients and allow patients to both seek appropriate therapy and evaluate its efficacy in an efficient manner.

As regards possible ME/CFS serum biomarkers, activin B has recently been added to the list. Activin B was stated as a possible marker that could distinguish ME/CFS cases and healthy controls, showing that a higher median of activin B level is observed in healthy controls compared to ME/CFS patients [13]. However, more multi-centre studies demonstrating the previously described tendency with large participant cohorts are needed to acknowledge activin B as a sensitive and specific serum biomarker. Moreover, activin B also had a tendency to predict the severity of the symptoms in patients with ME/CFS. This finding was observed using weighted standing time (WST) severity classes and analysing them together with other serum biomarkers, for example activin A or follistatin [13].

Activins, members of the transforming growth factor β (TGF-β) superfamily, were first isolated from porcine ovarian follicular fluid and identified as activating factors for the release of follicle stimulating hormone [14]. Different additional roles have since been identified for these proteins, including broad and complex effects on cell growth and differentiation, regulation of embryogenesis, development of the reproductive system, wound healing, stem cell differentiation and regulation of immune response [15,16]. Activins are disulfide-linked dimeric molecules in structure composed of βA- and βA-subunits (activin A), βB- and βB-subunits (activin B), or βA and βB-subunits (activin AB) [14]. Activin A has long been known to be a critical regulator of inflammation and immunity, and similar roles are now emerging for activin B, with which it shares 65% sequence homology [17]. These molecules and their binding protein, follistatin, are widely expressed, and their production is increased in many acute and chronic inflammatory conditions. Synthesis and release of the activins are stimulated by inflammatory cytokines, Toll-like receptor ligands, and oxidative stress. So far, activin A has been the most extensively studied TGF-b family member while activin B has received relatively little attention compared with activin A. In many cases activin B shares several of the functions of activin A; however, it may also exert functionally distinct effects from those of activin A [18]. There are data available showing that activin B production is increased in some cell types in response to inflammatory stimuli, most notably hepatic stellate cells, pituitary cells, and microglial cells [19,20]. Activin B regulates cellular migration by inducing actin stress fiber formation. Activins have been found in most tissues including placenta, reproductive organs, bone marrow, and brain [21].

Some studies suggest that activin may be an important mediator involved in the inflammatory response in the brain. Activin in microglia was first demonstrated during meningitis [22], however the role of activin in the brain microglia cells has not been fully understood. The findings of Sugama et al., where they used cultured microglial cells and rat brain, strongly suggest that activin may have inhibitory functions for microglial activation [20]. Activin is produced when microglia becomes activated, and this process is a major part of neuroinflammation, which is one of main pathogenetical mechanisms in myalgic encephalomyelitis. Knowing the widespread localization of activins, it has been reported that in a variety of tissues activin controls numerous processes and conditions, including inflammation, osteoporosis, stimulation of follicle stimulating hormone release from the pituitary, folliculogenesis in the ovary, erythroid differentiation, nerve cell survival, and tumour and embryonic development [23,24]. The fact that activin B can promote a loss of muscle mass [25] and is involved in immune dysregulation point out that it could be involved in the pathogenesis of ME/CFS, considering that muscle weakness and pain are included in the diagnostic criteria [26].

## 2. Materials and Methods

In total 134 patients [42 males (23–76 years old) and 92 females (23–68 years old)] with clinically diagnosed ME/CFS corresponding to 1994 Fukuda criteria and 54 healthy controls [41 males (18–65 years old) and 13 females (18–61 years old)] were recruited for this study. Healthy blood donors were included in the study as a control group. Plasma samples were always processed and stored according to the following standards: samples were frozen in aliquots and stored at −80 °C until the analyses. Human activin B concentration in blood plasma samples was analysed using the commercially available ELISA kit (LifeSpan BioSciences, Seattle, WA, USA). The assay measures ‘total’ activin B and is based on the sandwich ELISA principle. It is specific for the use of human plasma samples with the lower limit of detection being 15.63 pg/mL. All plasma samples were analysed in duplicate. The inter-assay and intra-assay CV were <6% and <6.15% respectively. Total duration of assay was 4 h. Briefly, all reagents, samples and standards were prepared and 100 μL was added to each well pre-coated with antibody specific for activin B. After an incubation of 1.5 h at 37 °C, 100 μL of biotin-conjugated antibody specific for activin B was added. Following an incubation of 1 h at 37 °C, 100 μL of an avidin-horseradish peroxidase conjugate was added and incubated for 30 min at 37 °C. Following this, 90 μL of tetramethylbenzidine substrate was added. This was followed by an incubation of 15 min at 37 °C. Lastly, 50 μL of stop solution was added and the plate was read at 450 nm using Microplate Reader Thermo Multiskan Ascent, USA.

The study design was approved by the Ethical Committee of Rīga Stradiņš University (Ethical code Nr.6-1/05/33 and date of approval 30.04.2020.) and written consent was obtained from all patients.

Between-group comparison was done by non-parametric Mann–Whitney U test or Kruskal–Wallis test followed by two-stage step-up method of Benjamini, Krieger, and Yekutieli as the post-hoc procedure. Statistical significance was set at *p* < 0.05.

## 3. Results

### 3.1. Human Activin B Concentration

**Figure 1:**
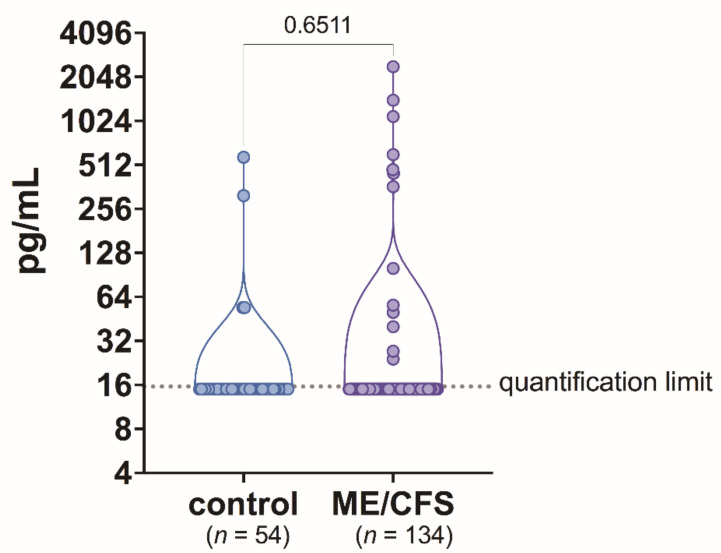
Activin B level in blood plasma samples of patients with ME/CFS and control group individuals.

Human activin B concentration in plasma samples was above 15.63 pg/mL in 13 out of 134 (9.7%) patients with ME/CFS and in 4 out of 54 (7.4%) healthy controls. For most patients—121 (96.69%)—and also most controls—50 (92.6%)—activin B concentration was below 15.63 pg/mL. Mean activin B concentration was compared between the ME/CFS patients and healthy controls. The study results did not show any statistically significant difference in activin B plasma level between ME/CFS cases and healthy controls (*p* = 0.6511) (Figure 1).

**Figure 2:**
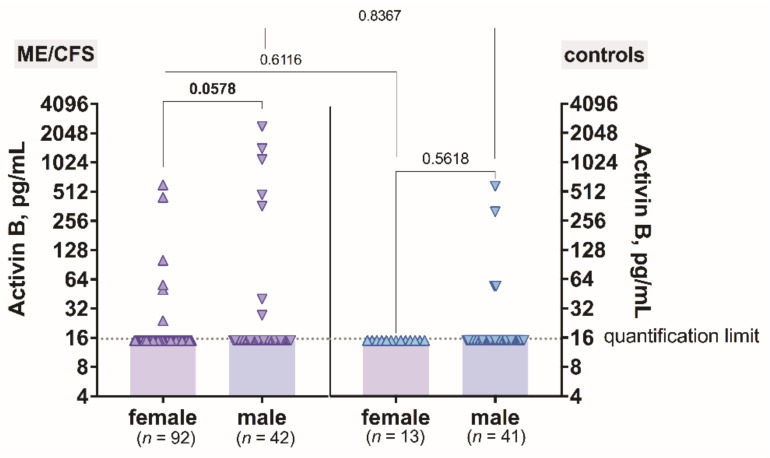
The levels of activin B in patients with ME/CFS and controls plotted against gender.

The level of activin B plotted against gender did not show any differences between male and female among ME/CFS patients (*p* = 0.0578) (Figure 2). Additionally, in the control group, there were no differences in activin B concentration between males compared to females (*p* = 0.5618) (Figure 2). When analysing the gender differences in activin B levels in ME/CFS patients compared to control men and women, no difference was found (*p* = 0.8367 and *p* = 0.6116 respectively) (Figure 2).

**Figure 3:**
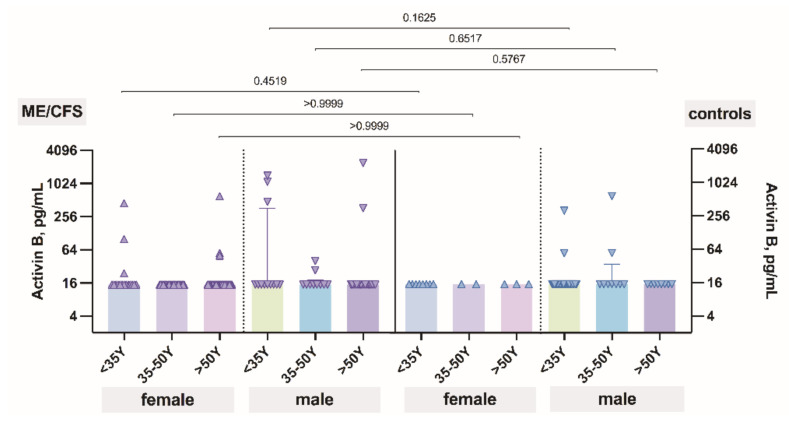
The levels of activin B in patients with ME/CFS and controls plotted against age.

The level of activin B plotted against age did show that activin B level does not change regarding the aging process of patients with ME/CFS and also controls (Figure 3). Additionally, no differences were observed between patients and controls in all age groups.

### 3.2. Clinical Data of ME/CFS Patients with Human Activin B Level above and below 15.63 pg/mL

As to the clinical characteristics, apart from the Fukuda criteria, we also investigated additional symptoms and patterns of fatigue to both investigate the core symptoms in our cohort, as well as understand whether there are any differences in fatigue pattern regarding activin B level. To achieve the goal, patients were interviewed using adapted semi-structured interview questions created by Minnock, et al. [27]. The questions were structured in 6 sections: causes and triggers of fatigue; character of fatigue; current symptoms; comorbidities; solutions for fatigue; and its influence on work disability. Multiple answers were proposed for each question. We used the Athens Sleep Questionnaire [28], including problems in 6 or more of the following sleep-related questions: impaired sleep induction, awakening during the night, final awakening earlier than desired, insufficient total duration of sleep, impaired quality of sleep and impaired sense of well-being and/or physical or mental functioning during the day, as well as sleepiness during the day. Visual analogue scale (VAS), ranging from 0 to 10, was also measured for all patients. To compare the clinical differences in each section of our questions considering activin B level, we divided respondents into two groups—with activin B level above 15.63 pg/mL (activin B positive group) and activin B level below 15.63 pg/mL (activin B negative group).

As to the possible trigger of fatigue, the most prevalent was considered physical work (38%), sleep disturbances (24%), mental work (22%), diet problems (9%) and emotional stressors (7%) in the activin B positive group, compared to mental work (31%), physical work (28%), eating disturbances (13%), sleep disturbances (16%), co-morbidities (6%) and drugs (6%) in the activin B negative group. In both groups, fatigue had been prevalent at least for the last 6 months, but for the most part fatigue had been present at least for the last year (in 67% and 70% of patients in the activin B positive and activin B negative group, respectively). For all of the participants, fatigue was unrelieved by rest and almost all in both groups (83% and 61% in activin B positive and negative group, respectively) admitted that fatigue was constant throughout the day without any changes in severity depending on the time of the day. In the activin B positive group, fatigue was more severe in the evening (17%), compared to the activin B negative group, where it was reported as more severe in the morning (22%) or variable throughout the day (17%). All of the participants in both groups answered that there have been periods when fatigue had been persistent for several days, weeks or months without any explicable reason.

Comparing the most common clinical symptoms that the respondents had felt during the period of the last 6 months besides fatigue, in both groups the five most prevalent symptoms from 22 proposed in the questionnaire were comparable: headache (100%), myalgia (92%), difficulty concentrating (92%) and arthralgia (83%) in the activin B positive group and myalgia (96%), difficulty concentrating (83%) arthralgia (71%) and sleep disturbances (58%) in the activin B negative group.

Regarding the reduction in occupational, personal, social, or educational activities, all of the participants reported that the above-mentioned activities were affected because of fatigue. Most of them have resigned (50% in activin positive and 52% in activin negative group, respectively), have changed their duties or reduced their workload (25% in activin positive and 35% in activin negative group, respectively), but the minority have not changed anything in their occupational activities (25% in activin positive and 13% in activin negative group, respectively). All of the participants answered that they factor in fatigue when making future plans and that their family members and/or friends have noticed fatigue and have tried to talk about it to the respondents. Besides that, some differences were noted in terms of family history of fatigue—there were no known relatives suffering from fatigue in the activin B positive group, whereas 22% noted at least one relative with similar symptoms in the activin B negative group.

In the assessment of subjective solutions to reduce the symptoms of fatigue, in both groups the majority of respondents consider physical activity or sleep hygiene to be the most effective (54% and 50% in the activin B positive and negative group, respectively). Pharmaceutical drugs were considered to be effective in the activin B positive group (38%), comparing to the activin B negative group (17%). 33% in the activin B negative and 8% in the activin B positive group have not found any solutions to their symptoms.

**Figure 4:**
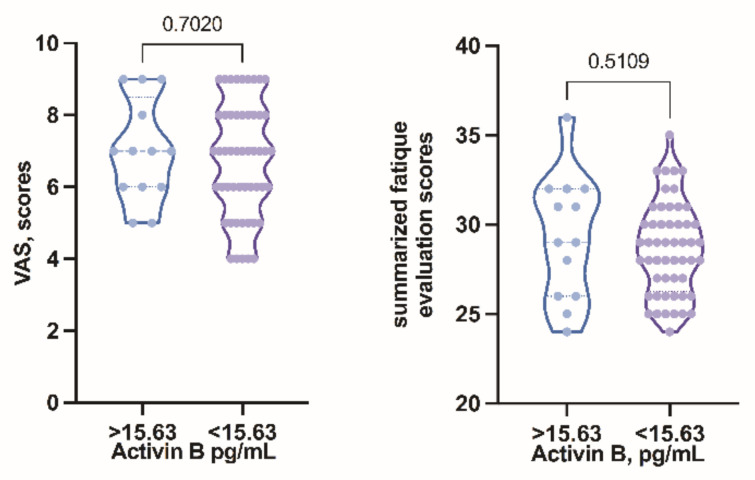
Symptom severity grading score compared in activin B positive and activin B negative groups.

VAS was measured for all patients and correlation between VAS and activin B level was conducted. Comparing the mean VAS score, it was 7 in both activin B positive and activin B negative groups. Apart from that, the scores were also equally distributed among both groups (Figure 4, showing no significant differences).

To be able to statistically compare the subjective severity of the clinical symptoms in both groups we decided to grade the answers from the questionnaire, taking into account the symptom count, the duration of fatigue (the longer the period of fatigue, the higher the grade), the impact of fatigue on quality of life, the pattern of fatigue and whether any solutions to ease the symptoms were found. Although the graph shows variations in distribution between groups, the obtained results did not show significant differences in subjective fatigue severity scores in activin B positive and negative groups (Figure 4).

**Figure 5:**
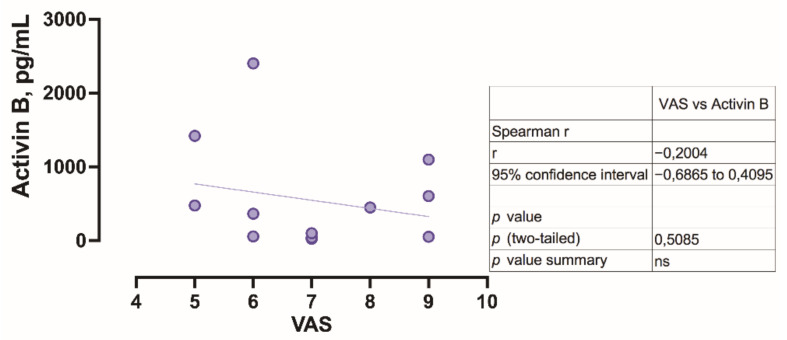
Correlation of visual analogue scale with activin B level.

Regarding the level of activin B, it was found to decrease with increasing VAS score (Figure 5); however no statistical significance was observed (*p* = 0.5085).

## 4. Discussion

Although ME/CFS has been under investigation for more than 30 years, progress on the examination of the diagnostic potential of serum biomarkers has not been rapid. Trusty serum biomarkers for ME/CFS are essential and necessary for this disabling and complex disease. According to the available data in the literature so far, the role of activin B in ME/CFS has been studied only by one group of researchers from The National Centre for Epidemiology and Public Health, Australia where 45 ME/CFS cases and 17 healthy controls were analysed [26]. By comparing ME/CFS patients with healthy controls, the researchers concluded that a statistically significant increase in activin B level is found in those with ME/CFS (*p* < 0.0001). Despite the relatively small sample size analysed, the authors stated that activin B could help to distinguish ME/CFS patients from those without the condition. Furthermore, the same group of scientists a few years later published a study with an increased ME/CFS group of 85 cases and concluded that activin B level showed a statistically significant decrease in those diagnosed with ME/CFS [13]. Based on data available in the literature about activin B in cases of ME/CFS, we tested the diagnostic potential of activin B by directly comparing 134 cases of ME/CFS with 54 healthy controls. In this study there was no statistically significant difference found regarding the level of activin B between ME/CFS cases and healthy controls, thereby limiting the use of activin B in the diagnostics of ME/CFS. The obtained study results show that the concentration of activin B in patients with ME/CFS and healthy controls do not significantly differ between male and female (*p* = 0.0578; *p* = 0.5618, respectively). Previously published data also show no significant difference between healthy male and female serum samples. However, significant differences were found between healthy values and the other groups (women undergoing IVF procedures, men with marked semen abnormalities and other) of the same gender [29]. Our results demonstrate that there were no age-related differences of activin B concentration in healthy adult males. Ambiguous data have been published showing that some studies did not show a difference in activin B levels between healthy men in the age group [29] while others showed an increase in activin B levels in older men [30].

Data published so far show a tendency for activin B to decrease with increasing age of a healthy adult female [29]. That may be associated with a decrease in the number of antral follicles in the ovaries with age, as the antral follicles have been found to express the βB subunit of activin B [31,32]. In this study, the results show that there was no relationship of activin B concentration with age in healthy adult females.

The clinical relevance of activin B has not been clear in ME/CFS; however, the correlation between VAS and activin B level was conducted. The concentration of activin B decreases with increasing VAS score (*r* = −0.2004); however no statistical significance was observed (*p* = 0.5085).

Many factors regulate activin B bioactivity but follistatin is considered as the major regulator [33]. As activin B can bind to follistatin it is very important to choose an assay which measures total activin B, not only “free” activin B, which is unbound to binding protein such as follistatin. The assay we choose is appropriate, because it measures total activin B. The strength of our study is the sufficient number of patients analysed. A limitation of our study can be considered the fact that no simultaneous comparative evaluation of activin B′s closely related sister molecule, activin A, which is also a protein associated with inflammation and tissue stress, has been performed.

Although different ME/CFS diagnostic criteria were used (Canadian consensus criteria in the study mentioned below [26] and Fukuda criteria in our study), comparing the clinical symptoms in the activin B positive group to the ones reported in the cross sectional study regarding activin B [26], the respondents apart from post-exertional fatigue present with difficulty concentrating and sleep disturbances in both cohorts, although respondents in our study were more prone to have myalgia, arthralgia and headache (compared to less than 10% in the study mentioned [26]). There were no patients presenting with new allergies or arrhythmias in our study, compared to approximately 50% of study participants having these symptoms in the other cohort. As stated above, activin B has the potential role to induce muscle wasting and pain [25]. Nevertheless, one of the core symptoms in the activin B positive group was myalgia, reported by 92%. The fact that 96% reported having it in the activin B negative group may imply that activin B does not influence the clinical presentation of muscle pain in ME/CFS patients. This finding is also substantiated by the fact that the VAS score did not show any statistically significant differences in both groups and even tends to decrease in the activin B positive group. Although we did not find any significant differences in clinical presentation of symptoms in both activin B positive and negative groups, more reports comparing the symptoms in both groups would be needed.

## 5. Conclusions

Results obtained in our study do not agree with the results of previously published works and do not confirm the clinical applicability of activin B as a biomarker for ME/CFS. Clinically, the respondents with increased activin B levels showed a tendency to have a lower VAS score, although more data is needed to evaluate the correlation.

## Data Availability

The datasets used and/or analyzed during the current study are available from the corresponding author on reasonable request.
